# Methods of Estimating Functional Efficiency of Liver

**Published:** 1915-12

**Authors:** 


**Methods of Estimating Functional Efficiency of Liver,** C. B. Van Zant, Denver, Col. Med., Nov.

1. Tests based on bile-forming function. (a) Bile is found in urine in all cases of jaundice due to hepatic disease (and obstruction not involving liver. Ed.), often when jaundice is not present, and in rapid haemolysis. Malaria, chloroform and ether poisoning, and organic diseases of liver, as acute yellow atrophy. Brule affirms that when single biliary constituents and not bile as a whole are eliminated in the urine, there must be organic lesion or disease of the liver cells.

1. (b). Hypercholesterinaemia. Henes believes that gall stones originate in the gall bladder when cholesterin exceeds 2.8 : 1000 in the blood. Such an excess may exist in fever without time for their formation, but otherwise the excess of cholesterin differentiates gall stones or gall bladder disease from duodenal and gastric ulcer and cancer, appendicitis, intestinal adhesions, etc.

1. (c). Phenoltetrachlorphthalein test. (Rowntree & Whipple) Normally, 400 m. g. injected into a vein is excreted in the bile and appears in the faeces within 48 hours, none appearing in the urine. Less than 30% of the dose administered, in the faeces or any appearing in the urine, indicates disease of the liver. Injuries, chloroform, phosphorus or other poisons, hepatic carcinoma and syphilis, obstructive jaundice are among the particular diagnostic indications.

1. (d). Urobilin and Urobilinogen. (Wilbur and Addis, Steiger, Labre, Chesney ,Marshall, Rowntree, von Jaksch) Normally all urobilin formed in the intestine is destroyed or changed into bile pigment. In almost any disturbance of the liver, except Pick's dieases (frosted liver) and chronic passive hyperaemia, one or both of these substances are found in the urine.

2. Tests based on urea-forming function. The normal liver changes ammonium compounds and amino-acids into urea, at least to a large extent. Thus, a reduction of urea in the blood and urine and an excess of amino-acids is theoretically a basis for estimating liver function. MacAdam holds that both absolute increase and increase relative to total N of the urine, are significant. Steiger believes that the determination of the amino acid output in the urine is the only practical method. Chesney, Rowntree and Marshall corroborate this view. Opie and Alford, Krehl and Landois corroborate the old view that crystals of leucin and tyrosin in the urine, along with jaundice, indicate disintegration of liver tissue as in acute yellow atrophy, late chloroform and phosphorus poisoning, certain toxaemias of pregnancy. Labbé and Bonchage consider that an excess of amino-acids and ammonia in the urine in diabetes allows us to incriminate the liver. Falk and Saxl hold that the diminution of urea and excess of ammonia and amino-acids in the urine, do not occur in leucocythaemia, amyloid degeneration, sarcoma nor chronic passive congestion; that these tests often occur in cancer and phosphorus poisoning; occasionally in catarrhal jaundice, gall stones, fatty degeneration and syphilis; very constantly and markedly in cirrhosis.

3. Tests based on sugar-forming function. (a) Strauss laevulose test. 100 grams of laevulose (It has been suggested that on account of the expense of c. p. laevulose, honey may be used. Ed.) ingested at once, should not produce laevulosuria. If it does occur, hepatic failure is suggested, the test being especially valuable in cirrhosis and chronic passive congestion.

3. (b) Bauer galactose test. 40 grams administered fasting in the morning should not produce galactosuria (samples being collected every 2 hours.) The test is constant in jaundice, frequent in gall stones, cirrhosis, cancer, etc. Wagner and Hatiegan consider it more dependable than the laevulose test.

4. Tests based on fibrinogen-forming function. The reduction of coagulation, as by the Whipple method, is noted in practically all severe hepatic diseases.

5. Tests based on adequacy of anti-thrombin-forming function. Denny and Minot have established the general principle but the test is not yet on a practical basis.

6. Tests based on ferment-forming or ferment-inhibiting function. (a) Ghedini's diastase test. Normal livers add diastase and, therefore maltose, to the blood. In hepatic disorders, the amount is lessened or nil.

6. (b) Whipple's lipase test. (inhibition) Lipase is increased 6-8 times in the blood in nearly all cases of eclampsia, liver injuries, chloroform intoxication, infections, acute yellow atrophy, cholangitis and liver abscess. In cirrhosis and obstructive jaundice, the test is not so significant.

6. (c). Haemoconiasis test. (Jeannin and Levant) The small refractive bodies normally seen in a drop of fresh blood are much increased after the ingestion of fat, 50 grams of butter being used, if the liver is normal. In hepatic disease, this increase does not occur and in serious disturbances as acute yellow atrophy the haemoconiae are reduced to 15-20 per drop. The authors cited have found this test especially useful in differentiating between mild and severe forms of jaundice in pregnancy. (Note: We would conjecture that this test should not be classified under this general heading, but that it depends upon the absorption of fats, under the influence of the bile salts. Ed).

6. (d). Fibrinolytic ferment test. (Goodpasture) A fibrin-dissolving ferment seems to be secreted only in atrophic cirrhosis. Blood drawn ante- or post-mortem, clots normally (but see 4 and 5) but the clot completely dissolves in 4-5 hours.

7. Tests based on the iron-storing function of the liver have not been devised, except as one may so consider the clinical observation of certain cases of pernicious anaemia.

8. Tests based on the protective function of the liver, in detoxicating certain nitrogenous substances, have not been devised. Absence of the detoxicating function may be the state in eclampsia, uraemia and possibly in cystinuria and alkaptonuria.

9. Acidosis test. There is some ground for the belief that the liver is the organ in which the acetone bodies are formed. The formation and appearance of these bodies in the blood and urine varies directly with liver function, not inversely as for most of the other tests.

Note. While this article may prove somewhat disappointing to those considering the subject merely as a means of securing diagnostic tests, we have given an unusually full abstract, as it deals collectively and systematically not only with actual tests, but with the general principles on which all such tests are to be judged and by means of which newer tests are to be developed. We have never seen so broad a discussion of these principles, expressed in simple terms.

